# 

**Published:** 1904-12

**Authors:** 


### ATLANTA JOURNAL-RECORD OF MEDICINE.

Successor to Atlanta Medical and Surgical Journal, Established 1855, and Southern Medical Record, Established 1870.

VOL. VI. ATLANTA, GA., DECEMBER, 1904. No. 9.

